# Transitional Lesions, One More Step Towards Understanding the Pathogenesis of Adenomyosis

**DOI:** 10.3390/jcm14134578

**Published:** 2025-06-27

**Authors:** Emilie Wacheul, Marie-Madeleine Dolmans, Jérôme Ambroise, Jacques Donnez, Alessandra Camboni

**Affiliations:** 1 Gynecology Research Unit, Institut de Recherche Expérimentale et Clinique, Université Catholique de Louvain, 1200 Brussels, Belgium; 2 Anatomopathology Department, Cliniques Universitaires Saint-Luc, 1200 Brussels, Belgium; 3 Gynecology Department, Cliniques Universitaires Saint-Luc, 1200 Brussels, Belgium; 4 Centre des Technologies Moléculaires Appliquées (CTMA), Institut de Recherche Expérimentale et Clinique, Université Catholique de Louvain, 1200 Brussels, Belgium; 5 Society for Research into Infertility, 1150 Brussels, Belgium

**Keywords:** adenomyosis, pathogenesis, transitional lesions, immune cells, mast cells

## Abstract

**Background/Objectives:** Adenomyosis is a benign gynecological disorder associated with abnormal uterine bleeding, dysmenorrhea, and subfertility. Its pathogenesis has not yet been elucidated. The most widely accepted theory points to repeated mechanical or hormonal stress at the endometrial–myometrial interface, leading to activation of the tissue injury and repair (TIAR) mechanism. Studies suggest that the immune system may play a role in disease pathogenesis, but inconsistencies persist due to differences in studied samples and evaluated menstrual cycle phases. The goal of our study was to apply a novel technique (multiplex) to investigate different immune cell phenotypes in uteri from adenomyosis patients according to the cycle phase. **Methods:** This study analyzed immune cell populations in adenomyotic uteri using immunohistochemistry and multiplex immunofluorescence on 30 adenomyotic and 15 healthy hysterectomy samples. **Results:** Compared to eutopic endometrium, transitional and adenomyotic lesions displayed reduced immune infiltrates, particularly T cells, NK cells, B cells, macrophages, and dendritic cells. Conversely, mast cells were significantly elevated in transitional lesions. **Conclusions:** The present study suggests mast cell implication in adenomyosis development and pain, through their implication in tissue remodeling, angiogenesis, and neurogenic inflammation. Transitional lesions highlighted the progressive nature of adenomyosis, supporting the TIAR hypothesis. These findings emphasize the importance of mast cells in disease progression and underscore the need for further studies to explore immune-targeted therapies.

## 1. Introduction

Adenomyosis is a benign gynecological disorder associated with abnormal uterine bleeding, dysmenorrhea, and subfertility. Histologically, it is defined as foci of glands and stromal cells invading the myometrium by more than 2.5 mm, surrounded by hyperplastic smooth muscle [1,2]. Its pathogenesis has not yet been elucidated. The most widely accepted theory points to repeated mechanical or hormonal stress at the endometrial–myometrial interface, leading to activation of the tissue injury and repair (TIAR) mechanism, subsequently triggering cellular and molecular responses that remodel the junctional zone [3]. This process facilitates the invagination of endometrial tissue into the myometrium. A number of studies suggest that the immune system may play a role in disease pathogenesis [4,5], but inconsistencies persist due to differences in studied samples and evaluated menstrual cycle phases. The goal of our study was to apply a novel technique (multiplex) to investigate different immune cell phenotypes in uteri from adenomyosis patients according to the cycle phase.

## 2. Materials and Methods

### 2.1. Study Participants and Tissue Collection

Human tissues used in this study were obtained in accordance with the Declaration of Helsinki and approved by the Ethics Committee of the Cliniques Universitaires Saint-Luc (CUSL) and Université Catholique de Louvain on 31 August 2020 (ref: 2020/14AOU/410).

A total of forty-five hysterectomy samples, fixed in 4% formaldehyde and embedded in paraffin blocks for histological analysis, were collected from the anatomopathology archives of CUSL. The adenomyosis group comprised thirty patients diagnosed via magnetic resonance imaging or transvaginal ultrasound (TVUS), with histological confirmation from surgical specimens. Morphological Uterus Sonographic Assessment (MUSA) criteria were used for the TVUS diagnosis of adenomyosis, and Bazot and Darai’s classification for the MRI diagnosis [6].

The healthy control group consisted of fifteen patients with no signs of adenomyosis, endometriosis, or any endometrial pathology. They had a hysterectomy for uterine fibroids or prolapsus (see Table 1). Samples were selected based on menstrual phases, with one-third derived from each phase. 

All patients were premenopausal and had not received hormones or selective steroid receptor modulators for at least three months prior to the intervention. 

### 2.2. Immunohistochemistry

Neutrophils, B cells, and mast cells were immunostained against specific surface receptors: CD15, CD20, and CD117, respectively.

Serial Sections (5 µm) were cut from each paraffin block using a microtome. After deparaffinization and rehydration, sections were incubated for 20 min in a 3% H_2_O_2_ solution to inhibit endogenous peroxidase activity. For heat-induced epitope retrieval (HIER), slides were heated in Tris-EDTA (pH 9) in a microwave for 20 min. Following cooling, 5% BSA was applied to block non-specific protein binding sites. Slides were then incubated at room temperature for one hour with primary antibodies: CD15 (Mouse anti-human, 1:300, BD Pharmingen (San Diego, CA, USA), clone MMA), CD20 (Mouse anti-human, 1:200, Biocare Medical (Pacheco, CA, USA), clone L26), and CD117 (Rabbit anti-human, 1:800, Dako (Carpinteria, CA, USA), A4502).

After rinsing, slides were incubated with EnVision anti-rabbit (Agilent (Santa Clara, CA, USA) K4003) or anti-mouse (Agilent K4001) secondary antibodies for 60 min. Bound antibody complexes were visualized using diaminobenzidine (DAB) (Dako K3468) staining, followed by counterstaining with hematoxylin (Dako S3301). Finally, the slides were dehydrated and mounted.

### 2.3. Immunofluorescence

T cells (panel A, see Appendix A Table A1) and macrophages (panel B, see Appendix A Table A2) were analyzed using a multiplex fluorescence technique based on tyramide amplification with fluorophores, enabling simultaneous detection of various cell subtypes on the same paraffin slide [7].

T cell characterization involved CD3 (T cells), CD8 (cytotoxic T cells), T-bet (type 1 T-helper cells), and GATA-3 (type 2 T-helper cells), alongside NKp46 for natural killer cells detection. For macrophage characterization, CD68 (monocytic cells), CD86 (M1 macrophages), and CD163 (M2 macrophages) were included in the same panel, along with CD1a for dendritic cell identification. 

The same procedural steps as for immunohistochemistry were followed, except that the slides were incubated with a fluorochrome–tyramide reagent for 10 min instead of DAB. The sequence was repeated until all antibodies from each panel had been applied. Nuclei were counterstained with Hoechst (10 mg/mL, dilution 1:1000), and a DAKO fluorescence mounting medium was used for slide mounting.

Positive controls included tissue samples known to express the markers of interest, such as appendix for CD15, CD117, CD20, CD3, CD8, T-bet, CD68, CD86, CD163, tonsil for CD1a, lung cancer for NKp46, and placenta for GATA-3. Negative controls consisted of endometrial sections incubated with 1% BSA instead of primary antibodies.

A complete list of antibodies and specific experimental conditions is provided in Appendix A Table A1 and Table A2. 

### 2.4. Analysis

**Figure 1:**
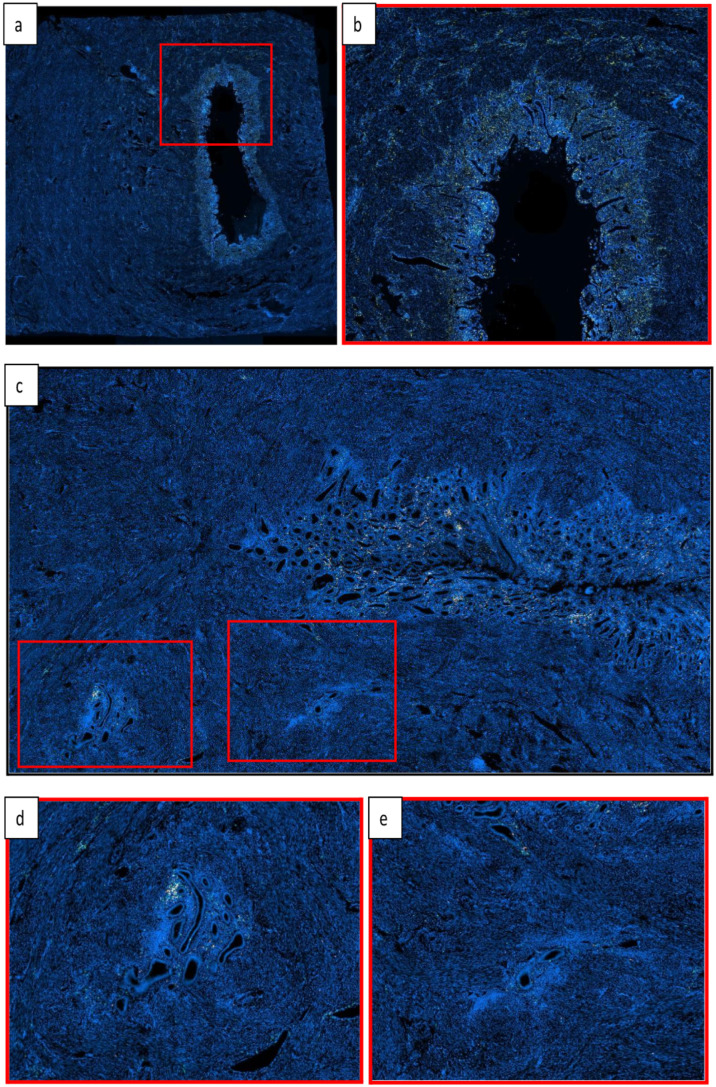
Microscopic comparison of healthy and adenomyotic uteri. This figure shows immunofluorescence staining with a multiplex technique to identify monocytic cells (**a**,**b**) and lymphocytes (**c**,**d**,**e**) on the same slide. (**a**) Histopathological image of a healthy uterus showing a regular and well-defined endo-myometrial junction (10× magnification). (**b**) Higher magnification view (20×) highlighting the sharply demarcated endo-myometrial junction in the healthy uterus. (**c**) Histopathological image of an adenomyotic uterus displaying an irregular and disrupted endo-myometrial junction. (**d**,**e**) High-power views of the adenomyotic uterus. These images reveal ectopic endometrial glands and stroma, separated from the eutopic endometrium and surrounded by hyperplastic myometrium. (**d**) The lesion is located at a depth exceeding 2.5 mm, corresponding to an adenomyotic lesion. The inflammatory infiltrate observed in the lesion appeared to be less dense than that seen in the eutopic endometrium. (**e**) The lesion is situated below 2.5 mm within the myometrium, classified as a “transitional lesion”. The inflammatory infiltrate observed in the lesion appeared to be less dense than that seen in the eutopic endometrium.

**Figure 2:**
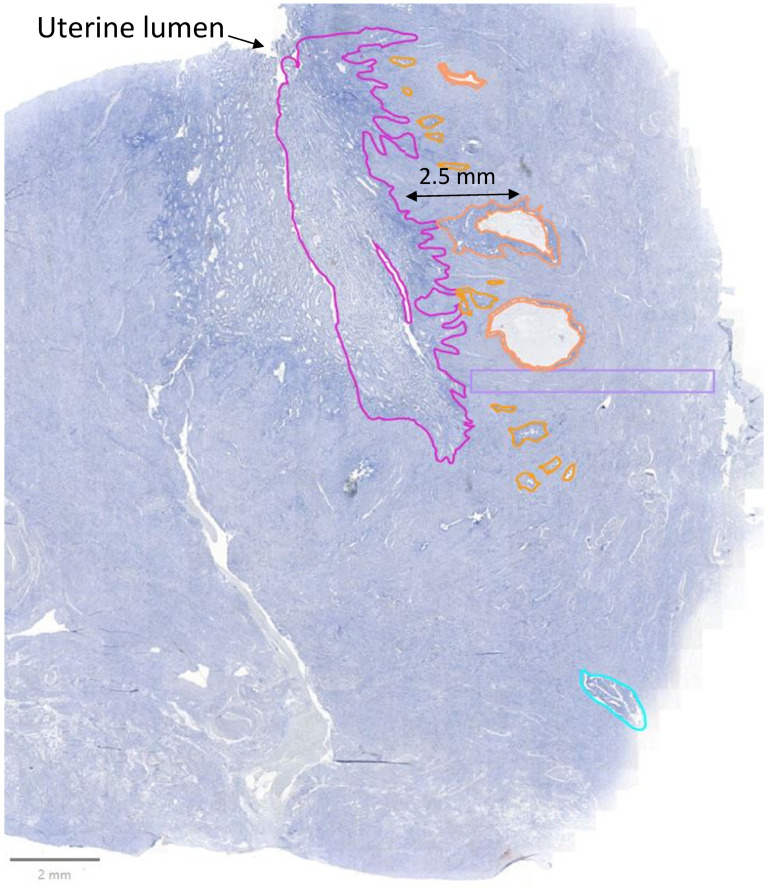
Isolation of areas of interest in an adenomyotic uterus. Eutopic endometrium showing an irregular and disrupted endo-myometrial junction (pink), myometrium (violet), adenomyotic lesions located > 2.5 mm from the endomyometrial junction (blue), and transitional lesions located < 2.5 mm from the endomyometrial junction (orange).

Whole sections were digitized using the Zeiss Axioscan Z1 (Zeiss, Oberkochen, Germany). Analyses were performed using QuPath 0.5.1 software. In the control group, we separated manually the endometrium (called “healthy endometrium”) from the myometrium. In the study group, sections were divided into areas of interest, including eutopic endometrium (called “disease endometrium”), myometrium, adenomyotic lesions, and transitional lesions, as explained further. The endometrium was defined as the continuous glandular surface composed of glands and stromal cells. Adenomyotic lesions were defined as foci of glands and stromal cells invading the myometrium by more than 2.5 mm from the junctional zone. Transitional lesions were characterized as foci separated from eutopic endometrium, but invading the myometrium by less than 2.5 mm (see Figure 1 and Supplementary Materials Figure S1). Areas were manually encircled, and classification was double-checked by two pathologists (see Figure 2). Using the “Cell Detection” tool in the QuPath software, we developed a script to identify individual cells and their corresponding nuclei within areas of interest. Optimization was performed on Hoechst-stained images, with various parameters adjusted—including pixel size, nuclear size, and cell expansion—to achieve a satisfactory balance of sensitivity and specificity (see Supplementary Materials Figure S2). For the detection of cells labeled with specific antibodies, we employed the “Single Measurement Classifier” tool to establish intensity thresholds for each fluorochrome individually. These thresholds were eye-calibrated to distinguish positive cells based on the desired signal intensity (see Supplementary Materials Figure S3). The quantity of inflammatory cells was calculated as the number of stained cells divided by the total number of cells in the area.

### 2.5. Statistical Analysis

Immune cell populations were quantitatively compared within the endometrium and myometrium, assessing differences between control subjects and patients with endometriosis and adenomyosis. These comparisons were performed using linear models within the limma (version 3.64.0) Bioconductor package. Furthermore, limma was employed to analyze differences in immune cell populations between ectopic lesions and eutopic endometrium within the endometriosis and adenomyosis cohorts. Statistical significance was determined after adjusting *p*-values using the Benjamini–Hochberg method, with an FDR threshold of 0.10. The threshold of 0.10 was used to refer to adjusted *p*-values (e.g., after multiple testing corrections) rather than raw *p*-values. All statistical computations were performed using R (version 4.5.0).

## 3. Results

### 3.1. Comparison of Endometrium Between Controls and DISEASE GRoups

**Figure 3:**
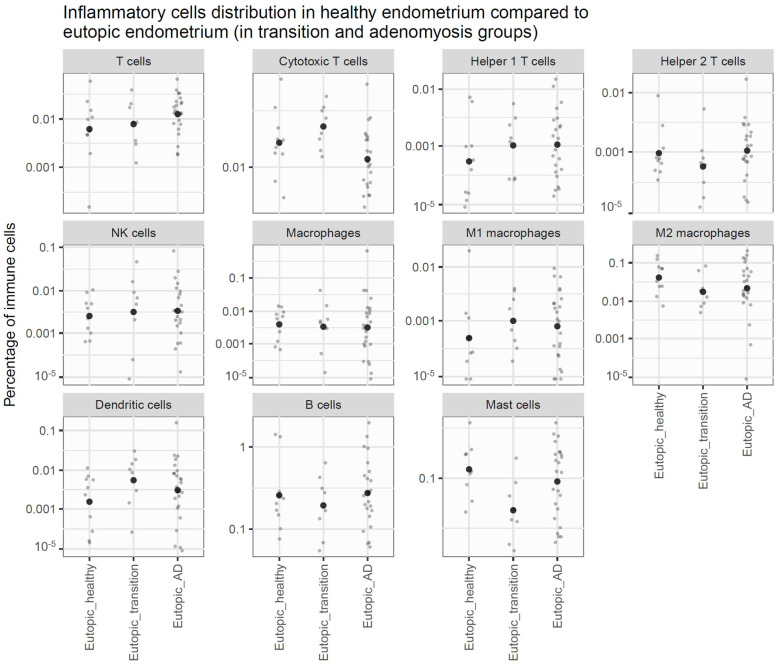
Inflammatory cell concentration in healthy endometrium compared to eutopic endometrium (in transitional lesions and adenomyotic groups). Graph showing the concentration of inflammatory cells in different areas studied. Each graph represents a cell type. Each dot represents a patient, and the large spot in the center shows the mean concentration of the group. There are no significant differences observed regarding the lymphoïde lineage. In the myeloïde lineage, we observed a significant decrease in the mast cell concentration in the disease endometrium of the transitional group.

In eutopic endometrium, no significant differences were observed in lymphoid lineage immune cell populations between healthy and disease groups. However, within the myeloid lineage, mast cell concentration was significantly decreased in the endometrium of the transitional group compared to healthy controls (logFC = −1.48; adjusted *p*-value = 0.028) (see Figure 3).

### 3.2. Comparison of Myometrium Between Controls and Disease Groups

No significant differences were observed in myometrial immune cell populations when comparing disease groups (adenomyosis and transition) to the control group.

### 3.3. Comparison of Lesions to Eutopic Endometrium Within Disease Groups

To investigate immune modifications during the development of adenomyosis lesions, we compared immune cell populations between lesions and eutopic endometrium in both disease groups.

**Figure 4:**
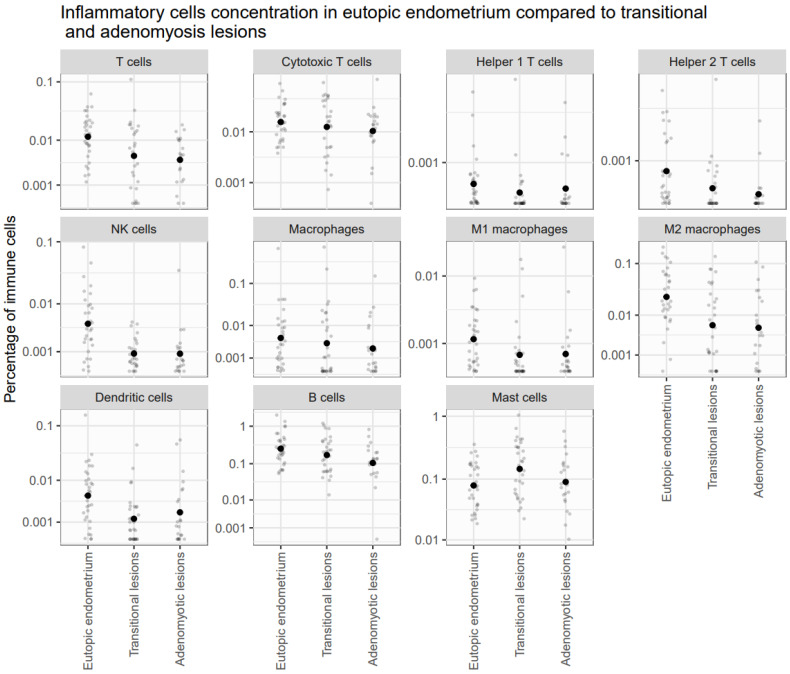
Inflammatory cell concentration in eutopic endometrium compared to transitional lesions and adenomyotic lesions. Graph showing the concentration of inflammatory cells in different areas studied. Each graph represents a cell type. Each dot represents a patient, and the large spot in the center shows the mean concentration of the group. We observed a general decrease in all immune cell types, except for mast cells, which exhibited increased concentrations in transitional lesions.

In the transitional group, significant modifications were observed between lesions and eutopic endometrium, including a reduced concentration of NK cells, dendritic cells, M1 and M2 macrophages, and helper 1 and 2 T cells. Conversely, mast cells appeared to be increased in the transitional lesions compared to the eutopic endometrium of the same group (logFC = 1.31; adjusted *p*-value = 0.048) (see Figure 4).

In the adenomyosis group, significant modifications were observed between lesions and eutopic endometrium, including a reduced concentration of T cells, NK cells, dendritic cells, M1 and M2 macrophages, B cells, and Helper 2 T cells (see Figure 4).

### 3.4. Phases of the Menstrual Cycle

All the analyses were performed with the phases merged. Indeed, when studying the interaction effect of the phases, it appears to not add information to the distribution of the immune cells.

## 4. Discussion

The identification of transitional lesions sheds light on their critical role and particularly the impact of mast cells on adenomyosis development. Mast cells may act as key facilitators by releasing inflammatory mediators like prostaglandins and cytokines, which promote tissue invasion through remodeling of uterine tissue, as has been demonstrated in endometriosis [8]. Furthermore, their secretion of vascular endothelial growth factor may contribute to enhanced angiogenesis, creating a supportive environment for the progression of adenomyotic lesions. As is well known, mast cells mediate neurogenic inflammation and pain. Anaf et al. identified an increased number of activated mast cells near the endometriosis lesions, located close to the nerve fibers [9]. Che et al. showed that activated mast cells may play a role in the pathogenesis of adenomyosis and particularly adenomyosis-related dysmenorrhea. In their study, the use of a drug that inhibits mast cell activation and suppresses mast cell degranulation (Mifepristone) relieved the dysmenorrhea symptom of adenomyosis patients by inhibiting the infiltration and the activity of degranulation of mast cells in eutopic and ectopic endometria [10].

Transitional lesions also underscore the dynamic nature of adenomyosis development, consistent with the TIAR mechanism’s emphasis on chronic injury and repair processes driving the condition’s progression [11]. These lesions likely form in original sites of microtrauma and repair, reflecting early invagination and supporting the hypothesis that adenomyosis develops progressively as a continuum rather than a binary condition.

## 5. Conclusions

Immunohistochemistry and immunofluorescence staining revealed a significantly reduced inflammatory infiltrate in both transitional and adenomyotic lesions compared to eutopic endometrium. This decline was observed across several immune cell types, including T cells, NK cells, B cells, macrophages, and dendritic cells. By contrast, mast cells appeared to be increased in transitional lesions compared to eutopic endometrium and adenomyotic lesions. No significant differences were noted in inflammatory cell populations of eutopic endometrium and myometrium between patients and healthy controls.

Our findings emphasize the need for further exploration of immune cell interactions, particularly within transitional lesions, to better understand the mechanisms underlying tissue invasion and immune evasion. Insights into these processes could provide valuable targets for early therapeutic interventions in adenomyosis.

## Figures and Tables

**Table 1:** Patient characteristics.

Pathology	Menstrual Phase	Age	BMI	Parity	Symptoms
Adenomyosis	Menstrual	46	30.1	G3P3	Menorrhagia and dysmenorrhea
Adenomyosis	Menstrual	49	28.5	G3P2	Menorrhagia and dysmenorrhea
Adenomyosis	Menstrual	43	37.9	G2P2	Menorrhagia
Adenomyosis	Menstrual	42	Not found	G4P4	Menorrhagia
Adenomyosis	Menstrual	48	Not found	G1P0	Menorrhagia
Adenomyosis	Menstrual	54	26.7	Not found	Not found
Adenomyosis	Menstrual	48	20.8	G1P1	Menorrhagia and dysmenorrhea
Adenomyosis	Menstrual	44	21.8	G2P2	Menorrhagia and dysmenorrhea
Adenomyosis	Menstrual	53	25.4	G2P2	Menorrhagia and dysmenorrhea
Adenomyosis	Proliferative	48	45.0	G3P3	Menorrhagia and dysmenorrhea
Adenomyosis	Proliferative	47	24.8	G2P0	Not found
Adenomyosis	Proliferative	50	31.6	Not found	Menorrhagia
Adenomyosis	Proliferative	46	28.3	G4P4	Menorrhagia and dysmenorrhea
Adenomyosis	Proliferative	42	30.3	G3P3	Menorrhagia
Adenomyosis	Proliferative	55	Not found	G5P3	Not found
Adenomyosis	Proliferative	43	Not found	Not found	Not found
Adenomyosis	Proliferative	51	30.8	G4P3	Menorrhagia
Adenomyosis	Proliferative	40	Not found	G3P3	Menorrhagia and dysmenorrhea
Adenomyosis	Secretory	47	Not found	G2P2	Menorrhagia and dysmenorrhea
Adenomyosis	Secretory	50	31.2	G4P3	Not found
Adenomyosis	Secretory	46	30.5	G5P2	Menorrhagia and dysmenorrhea
Adenomyosis	Secretory	37	40.1	G4P3	Menorrhagia and dysmenorrhea
Adenomyosis	Secretory	45	36.3	G1P1	Menorrhagia
Adenomyosis	Secretory	41	30.6	G6P5	Menorrhagia and dysmenorrhea
Adenomyosis	Secretory	45	Not found	G2P2	Menorrhagia
Adenomyosis	Secretory	42	20.6	G4P2	Menorrhagia and dysmenorrhea
Adenomyosis	Secretory	40	Not found	G3P2	Menorrhagia
Leiomyoma	Menstrual	39	21.1	G2P2	Not found
Leiomyoma	Menstrual	48	29.0	G6P4	Not found
Leiomyoma	Menstrual	44	23.7	G4P4	Not found
Leiomyoma	Menstrual	38	22.6	G4P4	Not found
Leiomyoma	Menstrual	42	Not found	Not found	Menorrhagia
Leiomyoma	Proliferative	44	50.2	Not found	Not found
Leiomyoma	Proliferative	42	28.7	G4P2	Not found
Prolapsus	Proliferative	56	20.8	GXP3	Not found
Leiomyoma	Proliferative	35	21.1	NA	Not found
Leiomyoma	Proliferative	49	39.0	G3P2	Menorrhagia
Leiomyoma	Secretory	41	24.3	G2P2	Menorrhagia
Leiomyoma	Secretory	37	Not found	G6P4	Menorrhagia and dysmenorrhea
Leiomyoma	Secretory	47	NA	G4PX	Not found
Leiomyoma	Secretory	40	28.3	G3P2	Menorrhagia and dysmenorrhea
Leiomyoma	Secretory	43	24.7	G3P2	Menorrhagia

## Data Availability

Data supporting reported results will be made available to the editors of the journal pre- and/or post-publication for review or query upon request.

## Supplementary Materials

The following supporting information can be downloaded at https://www.mdpi.com/article/10.3390/jcm14134578/s1, Figure S1: Microscopic comparison of healthy and adenomyotic uteri; Figure S2: Cell detection tool; Figure S3: Single measurement classifier.

## Appendix A

**Table A1:** Panel A.

Primary Antibody	Dilution	Antigen Retrieval	Secondary Antibody	Fluorochrome	Panel Position
GATA-3 (CST (Danvers, MA, USA) D13C9)	1/800	Citrate (pH 6)	Anti-rabbit	CF754	1
NKp46 (Abcam (Cambridge, UK) EPR22403-57)	1/100	Citrate (pH 6)	Anti-rabbit	CF555	2
T-bet (CST D6N8B)	1/300	EDTA (pH 9)	Anti-rabbit	AF594	3
CD8 (Dako clone C8/144B)	1/25	EDTA (pH 9)	Anti-mouse	CF488	4
CD3 (Invitrogen (Waltham, MA, USA) clone SP7)	1/150	Citrate (pH 6)	Anti-mouse	CF647	5

**Table A2:** Panel B.

Primary Antibody	Dilution	Antigen Retrieval	Secondary Antibody	Fluorochrome	Panel Position
CD86 (Abcam E2G8P)	1/100	EDTA (pH 9)	Anti-rabbit	CF488	1
CD1a (Agilent clone 010)	1/50	EDTA (pH 9)	Anti-mouse	CF754	2
CD163 (Sanbio Mob460-05)	1/40	Citrate (pH 6)	Anti-mouse	CF555	3
CD68 (Abcam ab955)	1/100	Citrate (pH 6)	Anti-mouse	CF647	4

Fluorochromes: Alexa Fluor Tyramide Reagent (Invitrogen): diluted 200-fold in borate buffer. CF Tyramide Reagent (ThermoFischer (Waltham, MA, USA)): diluted 1000-fold in borate buffer.
