# Supplementary material for: Transitional Lesions, One More Step Towards Understanding the Pathogenesis of Adenomyosis
Source: J Clin Med. 2025 Jun 27;14(13):4578. doi: 10.3390/jcm14134578 (PMC12249794; doi:10.3390/jcm14134578)
Supplement: Supplementary file 1 [file jcm-14-04578-s001.zip › jcm-3584200-supplementary.pdf]

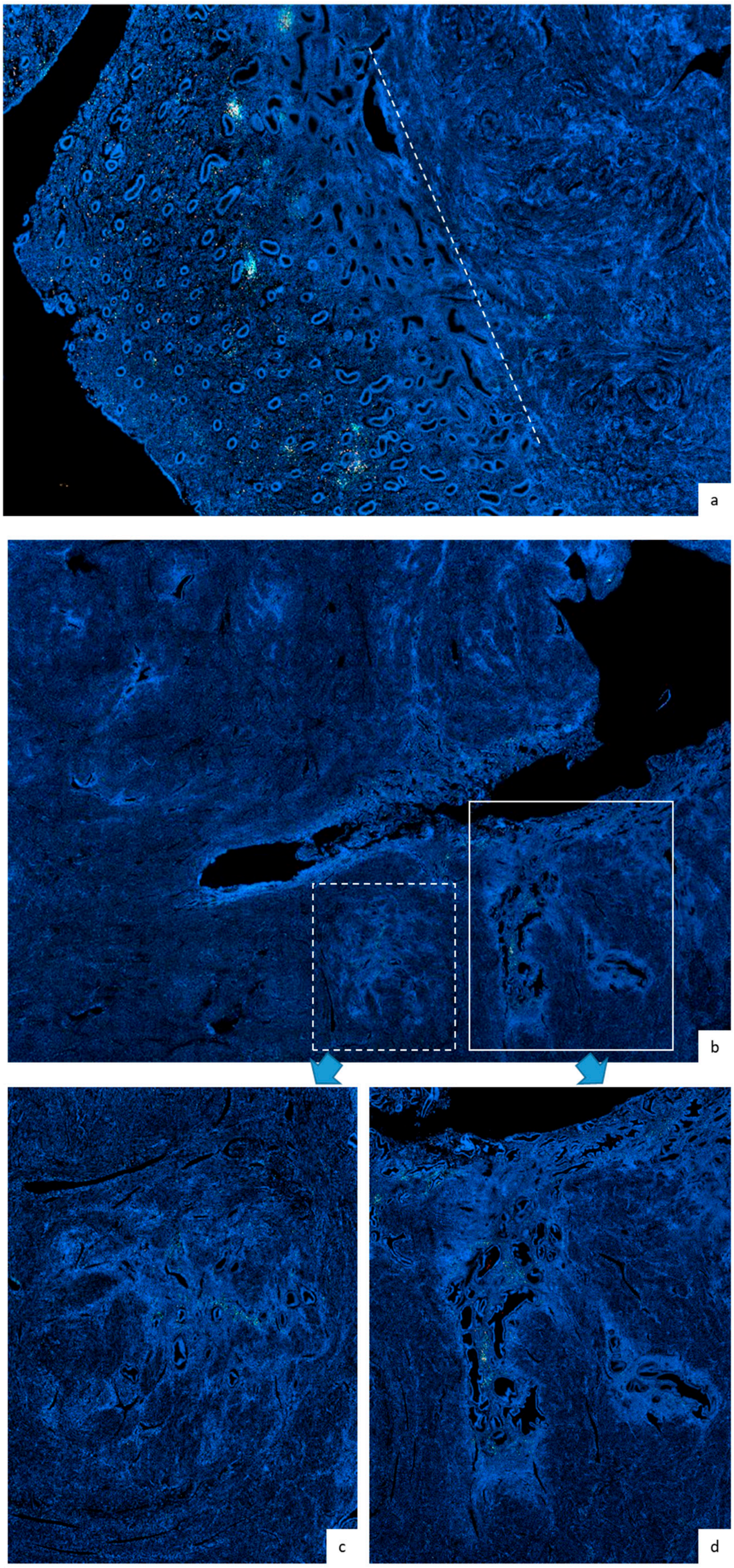

Figure S1. Microscopic comparison of healthy and adenomyotic uteri. This figure shows immunofluorescence staining using the multiplex technique to identify lymphocytes on the same slide. **a** Histopathological image of a healthy uterus

showing a regular and well defined endomyometrial junction (20x magnification). **b** Histopathological image of an adenomyotic uterus with ectopic endometrial glands and stroma surrounded by hyperplastic myometrium (10x magnification). An irregular and disrupted endomyometrial junction is visible on the right (inside square). **c** High-power view of the adenomyotic uterus focusing specifically on the transitional lesion (20x magnification). **d** High-power view of the adenomyotic uterus focusing on the irregular and disrupted endomyometrial junction (20x magnification).

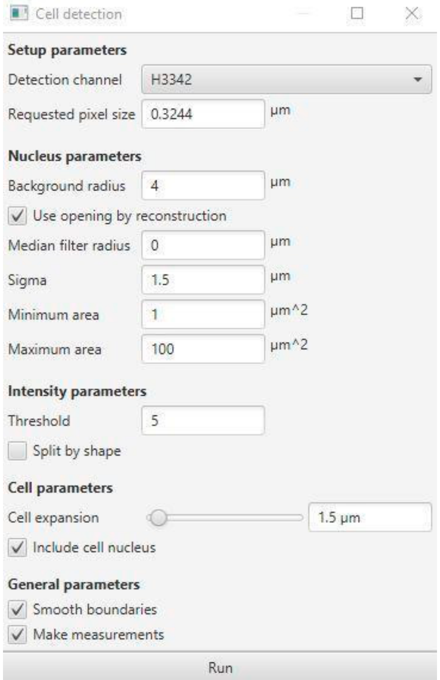

Figure S2. Cell detection tool.

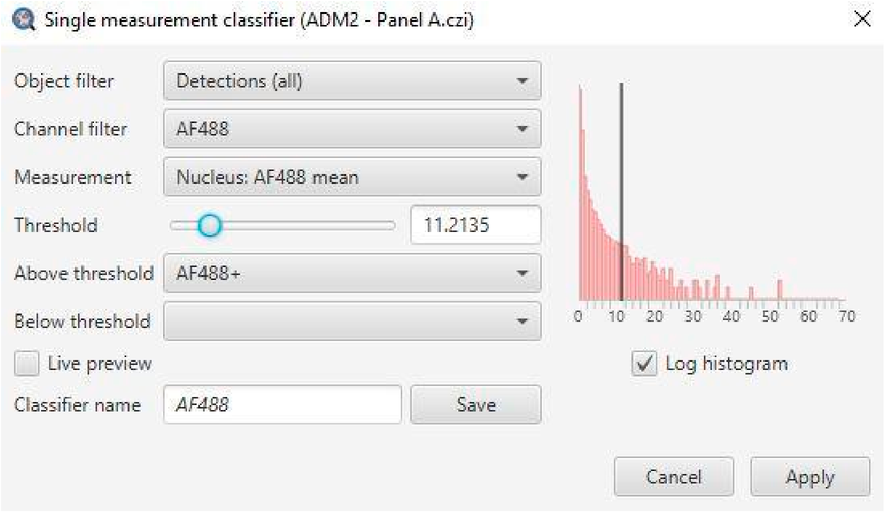

Figure S3. Single measurement classifier.
